# Spectrum of Bone Marrow Pathological Findings in Patients Undergoing Bone Marrow Biopsy: A Cross-Sectional Study

**DOI:** 10.7759/cureus.114122

**Published:** 2026-08-07

**Authors:** Fawad Mueen Arbi, Talha Irfan Khan, Haris Irfan Khan, Farina Farani

**Affiliations:** 1 Medicine and Surgery, Quaid-e-Azam Medical College, Bahawalpur, PAK; 2 Medicine, Shahida Islam Medical Complex, Lodhrān, PAK; 3 Anaesthesiology, University Hospital Limerick, Limerick, IRL; 4 Gynaecology, Bahawal Victoria Hospital, Bahawalpur, PAK

**Keywords:** acute, anemia, aplastic, bone marrow examination, cross-sectional studies, hematologic neoplasms, leukemia, myeloid

## Abstract

Introduction

Bone marrow biopsy is a diagnostic cornerstone in the evaluation of unexplained cytopenias and suspected haematological neoplasms; however, diagnostic frequencies vary widely across populations. This study describes the spectrum of bone marrow pathology recorded in a single-centre registry and explores, as a secondary exploratory aim, whether it varies by sex and age.

Methods

This retrospective cross-sectional study analysed 181 bone marrow examination records, which constituted the unit of analysis; no patient identifiers were available to confirm unique-patient status. Diagnoses were classified into 13 registry-derived categories using a prespecified, fully documented classification dictionary and subsequently collapsed into seven broad groups. The association between sex and diagnostic category was assessed using the chi-square test and the Fisher-Freeman-Halton exact test because of sparse cell counts. Age was compared across diagnostic groups using the Kruskal-Wallis test as the prespecified primary analysis because of non-normality, with ANOVA performed as a sensitivity analysis, followed by Dunn’s post hoc test.

Results

The median recorded age was 30 years (IQR, 20-50 years), and 66.9% of the records were from males. Acute leukaemia was the most frequent diagnosis (49.2%; 95% CI, 42.0%-56.4%), followed by aplastic anaemia/bone marrow failure syndrome (14.4%), inconclusive/deferred opinions (12.2%), non-neoplastic/reactive marrow (8.3%), myelodysplastic/myeloproliferative overlap (6.1%), lymphoproliferative/plasma cell neoplasms (6.1%), and other diagnoses (3.9%). Sex was not statistically significantly associated with diagnostic category (χ² = 5.995, df = 6, p = 0.424; exact p = 0.475; Cramér’s V = 0.182). Males accounted for the majority of records in most, but not all, groups; females constituted the majority in the non-neoplastic/reactive marrow group (53.3%). Age differed significantly across the groups (Kruskal-Wallis H = 40.12, df = 6, p < 0.001, epsilon-squared = 0.196). The myelodysplastic/myeloproliferative overlap group had the highest median age (62 years), whereas the aplastic anaemia/bone marrow failure syndrome group had the lowest median age (20 years).

Conclusions

Acute leukaemia predominated in this referral-heavy registry sample. Recorded age varied significantly by diagnostic category, whereas sex was not statistically significantly associated with diagnostic category. A substantial proportion of acute leukaemia records lacked a documented lineage subtype. Limitations related to the unit of analysis and sampling period are detailed in the text; therefore, the findings should be interpreted as descriptive and hypothesis-generating.

## Introduction

Bone marrow aspiration and trephine biopsy are indispensable investigations in clinical haematology, providing material for morphological, architectural, and immunophenotypic assessment that cannot be fully obtained from peripheral blood examination alone [1]. Trephine biopsy, in particular, preserves marrow architecture and cellularity, allowing the detection of focal lesions, fibrosis, and infiltrative disorders that aspirate cytology may miss [1,2]. Together, aspirate cytology, trephine histology, cytochemistry, and immunohistochemistry form a complementary diagnostic framework that underlies the modern classification of haematological disorders [3].

Patients are referred for bone marrow examination for a wide range of indications, most commonly unexplained cytopenias and suspected haematological malignancy [4]. The resulting diagnostic spectrum is correspondingly broad, encompassing reactive and nutritional causes of cytopenia, bone marrow failure syndromes, myelodysplastic and myeloproliferative neoplasms (MPNs), acute leukaemias, lymphoproliferative disorders, plasma cell neoplasms, and marrow infiltration by non-haematological malignancies [4,5]. The relative frequencies of these categories differ substantially among populations, reflecting differences in referral patterns, case mix (general medical versus oncology referral centres), and the age structure of the population studied [4-6]. Common clinical presentations prompting bone marrow examination include unexplained anaemia, pancytopenia, persistent leukocytosis or thrombocytopenia, unexplained fever, suspected acute or chronic leukaemia, lymphoma staging, plasma cell disorders, and suspected bone marrow failure syndromes.

Accurate subclassification of acute leukaemia and other haematological neoplasms increasingly depends on integrating morphology with immunophenotyping and, where available, cytogenetic and molecular data, as codified in successive revisions of the WHO classification of haematolymphoid tumours and the parallel International Consensus Classification [7-10]. In many resource-constrained or high-throughput diagnostic settings, however, a proportion of acute leukaemia cases are reported with a general diagnosis of “acute leukaemia” without complete lineage subtyping, which limits both individual patient management and the interpretability of registry-level data [2,11].

Despite the central role of bone marrow biopsy in haematological diagnosis, there remains a relative paucity of descriptive data characterising the full spectrum of bone marrow pathology encountered in routine diagnostic practice, rather than focusing on a single clinical presentation or disease category, such as pancytopenia or acute leukaemia, respectively [4-6]. The present study was therefore designed to describe the complete spectrum of bone marrow pathology in a consecutive series of bone marrow examination records from a single centre and to examine whether this spectrum varied by recorded sex and age.

## Materials and methods

Study design and setting

This was a retrospective, descriptive cross-sectional study based on a structured bone marrow examination reporting registry. Records were retrieved and de-identified before analysis. The data available to the analyst comprised registry entries recorded between June and December 2025 at Bahawal Victoria Hospital, an affiliated teaching hospital of Quaid-e-Azam Medical College. Because the registry timestamp reflected the date of data entry rather than a defined recruitment window, the study should be interpreted as a consecutive case series derived from routine diagnostic registry records at the reporting centre rather than as a fixed-calendar-period cohort.

Participants

All bone marrow aspirate and/or trephine biopsy records containing a final pathological opinion were eligible. No otherwise eligible records were excluded; records in which the pathological opinion was documented as inconclusive, deferred, or “none of the above” were retained and analysed as a distinct diagnostic category rather than discarded to avoid selection bias against non-diagnostic examinations. A total of 181 records met these criteria and formed the analytical dataset. The unit of analysis was the bone marrow examination registry record. Owing to the retrospective nature of the registry, patient-level verification of repeat examinations was not consistently possible; therefore, the findings should be interpreted as representing registry records rather than confirmed unique patients.

Variables and data cleaning

The extracted variables comprised demographic data (age and sex), referral indication, complete blood count parameters (haemoglobin, mean corpuscular volume (MCV), mean corpuscular haemoglobin (MCH), WBC count, and platelet count), peripheral blast percentage, aspirate findings (cellularity, dominant cell type, adequacy of haematopoiesis, and blast percentage), trephine findings (cellularity and architecture), a panel of immunohistochemical markers (CD34, CD117, MPO, PAX5, CD79a, CD3, TdT, and others where recorded), and the final pathological opinion.

Numeric fields contained inconsistent formatting, such as decimal commas, stray alphabetic characters, and threshold notation such as “<5%.” These fields were standardised programmatically: decimal commas were converted to decimal points, non-numeric characters were removed, and values reported as “<X%” were recoded as X/2 using a prespecified midpoint-substitution rule. One haemoglobin value (43 g/dL) was considered physiologically implausible and was treated as a data-entry error and recoded as missing rather than analysed at face value or arbitrarily corrected. Immunohistochemical markers were coded as binary variables (positive = 1 and negative = 0), with blank entries treated as not performed/missing.

The free-text pathological opinion was mapped to 13 mutually exclusive diagnostic categories using explicit, prespecified text-matching rules. For inferential analysis, these categories were further collapsed into seven broad groups to avoid sparse cells: acute leukaemia (all acute myeloid leukaemia (AML), B-lineage, T-lineage, and lineage-unspecified acute leukaemia records combined); aplastic anaemia/bone marrow failure syndrome; myelodysplastic syndrome (MDS)/MPN overlap; lymphoproliferative disorder/plasma cell neoplasm; non-neoplastic/reactive marrow; inconclusive/deferred; and a residual ‘other’ category combining infiltrative/infectious diagnoses, metastatic carcinoma, and unspecified chronic leukaemia. Sixty-five records were reported only as “acute leukaemia” without a documented lineage subtype. These were retained as a single unspecified-lineage subgroup within the acute leukaemia category rather than being reclassified by the study team using the same immunohistochemical markers that were subsequently tested for diagnostic associations, because doing so would have introduced circularity between the predictor and outcome.

The diagnostic categories used in this study represent registry-derived diagnostic labels based on the original pathological opinions and were grouped solely for descriptive and statistical purposes. They should not be interpreted as an independent WHO/ICC reclassification performed by the investigators.

In addition to demographic characteristics and complete blood count parameters, MCV (MCV), MCH, peripheral blast percentage, bone marrow morphological findings, and immunohistochemical marker results were extracted from the registry to support data verification and characterisation of the recorded diagnoses. These variables were not included in the descriptive baseline characteristics or inferential analyses because they were incompletely recorded across diagnostic groups and were not primary study outcomes.

Statistical analysis

Continuous variables were summarised as means ± SDs and, given evidence of skewness, as medians with IQRs. Categorical variables were summarised as frequencies and percentages. Missing data were handled using complete-case analysis for each specific test, and the number of records contributing to each analysis was reported alongside the corresponding result.

Sex was cross-tabulated against the seven broad diagnostic categories and analysed using Pearson’s chi-square test of independence. Because more than 20% of the expected cell counts were below 5, an exact test was also performed. Because exact enumeration can be computationally intensive for tables larger than 2 × 2, a Fisher-Freeman-Halton-type exact p-value was estimated using Monte Carlo simulation with 100,000 random tables generated under the fixed-margin null distribution using NumPy’s default_rng with a seed of 42. Cramér’s V was reported as the effect size.

Age was compared across the seven broad diagnostic categories using the Kruskal-Wallis H test, which was the prespecified primary analysis. The Shapiro-Wilk test indicated non-normality of the pooled residuals (W = 0.972, p = 0.001), and Levene’s test indicated unequal group variances (F = 2.78, p = 0.013). Classical one-way ANOVA was performed as a sensitivity analysis. When the omnibus test was significant, pairwise post hoc comparisons were performed using Dunn’s test with the Bonferroni correction for multiple comparisons. Epsilon-squared (ε²) was reported for the Kruskal-Wallis test, and eta-squared (η²) was reported for the classical ANOVA.

A two-sided p-value of <0.05 was considered statistically significant throughout. All analyses were performed using Python 3 with the pandas, SciPy, and scikit-posthocs libraries.

Ethical considerations

This study used retrospective, de-identified laboratory registry data and involved no direct patient contact or intervention. Ethical approval was obtained from the Institutional Review Board of Quaid-e-Azam Medical College (approval No. 2306/DME/QAMC Bahawalpur; July 7, 2025). This manuscript was prepared in accordance with the Strengthening the Reporting of Observational Studies in Epidemiology (STROBE) reporting guideline for observational studies [12]. The requirement for informed consent was formally waived because the study used retrospectively collected, de-identified registry data without direct patient contact.

## Results

Of the 181 records analysed, 121 (66.9%) were from males and 60 (33.1%) were from females. The mean age was 36.0 ± 18.9 years (median, 30 years; IQR, 20-50 years; range, 8-82 years). Baseline complete blood count parameters and their distributions across the broad diagnostic categories are summarised in Table 1.

**Table 1:** Demographic and clinical characteristics by broad diagnostic category (N = 181). BM: Bone marrow; Hb: Haemoglobin; MDS: Myelodysplastic syndrome; MPN: Myeloproliferative neoplasm; y: Years. One haemoglobin value (43 g/dL) was considered physiologically implausible and treated as missing; therefore, the overall haemoglobin summary was based on n = 180 available records.

Diagnostic Group	n (%)	Age, y (Mean ± SD)	Male, n (%)	Hb, g/dL (Mean ± SD)	WBC, ×10⁹/L (Mean ± SD)	Platelets, ×10⁹/L (Mean ± SD)
Acute leukaemia	89 (49.2)	33.0 ± 17.0	62 (69.7)	7.4 ± 2.0	43.0 ± 76.2	34 ± 38
Aplastic anaemia/BM failure syndrome	26 (14.4)	22.6 ± 8.6	18 (69.2)	5.9 ± 2.5	4.2 ± 10.6	22 ± 31
Inconclusive/deferred	22 (12.2)	41.2 ± 19.7	12 (54.5)	8.0 ± 2.6	12.0 ± 31.7	137 ± 138
Non-neoplastic/reactive marrow	15 (8.3)	35.1 ± 19.1	7 (46.7)	6.7 ± 2.0	4.6 ± 2.6	161 ± 171
MDS/MPN overlap	11 (6.1)	61.4 ± 16.9	8 (72.7)	7.3 ± 1.9	11.3 ± 9.6	235 ± 351
Lymphoproliferative disorder/plasma cell neoplasm	11 (6.1)	52.6 ± 14.1	9 (81.8)	8.5 ± 1.5	79.0 ± 195.2	130 ± 111
Other (infiltrative/infectious diagnoses, metastatic carcinoma, or chronic leukaemia)	7 (3.9)	46.2 ± 17.9	5 (71.4)	9.4 ± 1.1	110.0 ± 120.1	236 ± 190
Overall	181 (100)	36.0 ± 18.9	121 (66.9)	7.3 ± 2.2ᵃ	31.7±77.0	79.3±136.0

The complete 13-category diagnostic spectrum is shown in Table 2. Acute leukaemia was the single largest category overall, and within this category, the majority of records (65/89, 73.0%) had no documented lineage subtype.

**Table 2:** Spectrum of bone marrow pathological diagnoses (N = 181). ALL: Acute lymphoblastic leukaemia; AML: Acute myeloid leukaemia; MDS: Myelodysplastic syndrome; MPN: Myeloproliferative neoplasm.

Diagnostic Category	n	%
Acute leukaemia, lineage unspecified	65	35.9
Aplastic anaemia/bone marrow failure syndrome	26	14.4
Inconclusive/deferred/other opinion	22	12.2%
AML	16	8.8
Non-neoplastic/reactive marrow	15	8.3
B-cell ALL/B-cell lymphoproliferative disorder	7	3.9
Lymphoma/lymphoproliferative disorder	6	3.3
MDS/MPN overlap	6	3.3
MDS	5	2.8
Plasma cell neoplasm (multiple myeloma)	5	2.8
Metastatic carcinoma	3	1.7
T-cell neoplasm	1	0.6
Chronic leukaemia, unspecified	1	0.6

Chi-square analysis: sex and diagnostic category

The null hypothesis was that sex distribution was independent of the broad diagnostic category. Table 3 presents the contingency table and test results. Overall, males predominated in most diagnostic groups, but the proportion of records from males ranged from 46.7% in the non-neoplastic/reactive marrow group to 81.8% in the lymphoproliferative/plasma cell neoplasm group.

**Table 3:** Sex distribution across broad diagnostic categories with corresponding chi-square and exact analyses. BM: Bone marrow; MDS: Myelodysplastic syndrome; MPN: Myeloproliferative neoplasm.

Diagnostic Group	Female, n (Row %)	Male, n (Row %)	Total
Acute leukaemia	27 (30.3)	62 (69.7)	89
Aplastic anaemia/BM failure syndrome	8 (30.8)	18 (69.2)	26
Inconclusive/deferred	10 (45.5)	12 (54.5)	22
Non-neoplastic/reactive marrow	8 (53.3)	7 (46.7)	15
MDS/MPN overlap	3 (27.3)	8 (72.7)	11
Lymphoproliferative disorder/plasma cell neoplasm	2 (18.2)	9 (81.8)	11
Other (infiltrative/infectious diagnoses, metastatic carcinoma, or chronic leukaemia)	2 (28.6)	5 (71.4)	7
Total	60 (33.1)	121 (66.9)	181

There was no statistically significant evidence of an association between sex and broad diagnostic category (χ² = 5.995, df = 6, p = 0.424; Monte Carlo Fisher-Freeman-Halton exact p = 0.475; Cramér’s V = 0.182). Although males predominated in most groups, the findings did not provide statistically significant evidence that the relative distribution of diagnoses differed by sex.

Kruskal-Wallis analysis of age across diagnostic categories

The null hypothesis was that mean age did not differ across the seven broad diagnostic categories. The Shapiro-Wilk test indicated non-normality of the pooled residuals (W = 0.972, p = 0.001), and Levene’s test indicated unequal group variances (F = 2.78, p = 0.013). The Kruskal-Wallis test was the prespecified primary analysis, with classical one-way ANOVA performed as a sensitivity analysis.

Age differed significantly across the diagnostic groups (Kruskal-Wallis H = 40.12, df = 6, p < 0.001; ε² = 0.196). Classical one-way ANOVA also showed a significant difference (F = 10.13, df = 6, 174, p < 0.001; η² = 0.259) (Table 4). Dunn’s post hoc comparisons showed that records classified as MDS/MPN overlap and lymphoproliferative/plasma cell neoplasms involved significantly older individuals than records classified as aplastic anaemia/bone marrow failure syndrome. Records classified as MDS/MPN overlap also involved significantly older individuals than those classified as acute leukaemia. No other pairwise comparisons reached statistical significance after the Bonferroni correction.

**Table 4:** Analysis of age across broad diagnostic categories using Kruskal-Wallis and one-way ANOVA, with Dunn’s post hoc comparisons. The Kruskal-Wallis H test was the primary analysis, and classical one-way ANOVA was performed as a sensitivity analysis. Only statistically significant Dunn’s pairwise comparisons are presented (Bonferroni-adjusted p < 0.05). BM: Bone marrow; df: Degrees of freedom; MDS: Myelodysplastic syndrome; MPN: Myeloproliferative neoplasm; η²: Eta-squared; ε²: Epsilon-squared.

Test or Comparison	Statistic	df	P-value	Effect Size
Omnibus analyses				
Classical one-way ANOVA	F = 10.13	6, 174	<0.001	η² = 0.259
Kruskal-Wallis H test (primary analysis)	H = 40.12	6	<0.001	ε² = 0.196
Significant Dunn’s post hoc pairwise comparisons				
Aplastic anaemia/BM failure syndrome versus MDS/MPN overlap	-	-	<0.001	-
Aplastic anaemia/BM failure syndrome versus lymphoproliferative/plasma cell neoplasm	-	-	<0.001	-
Acute leukaemia versus MDS/MPN overlap	-	-	0.001	-
Aplastic anaemia/BM failure syndrome versus inconclusive/deferred	-	-	0.009	-
Acute leukaemia versus lymphoproliferative/plasma cell neoplasm	-	-	0.035	-
MDS/MPN overlap versus non-neoplastic/reactive marrow	-	-	0.041	-

## Discussion

In this consecutive series of 181 bone marrow examination records, acute leukaemia was by far the most frequent diagnostic category, accounting for almost half of all records once lineage-unspecified records were grouped with the documented AML, B-lineage, and T-lineage subgroups. This proportion is considerably higher than that reported in several published bone marrow spectrum studies from general medical or mixed-referral populations, in which acute leukaemia typically accounted for approximately 9%-28% of cases, with reactive, nutritional, or aplastic causes of cytopenia predominating instead [4-6,13,14]. For example, a 2024 Pakistani series of 120 patients with pancytopenia found megaloblastic anaemia in 40% of cases and aplastic anaemia in 16.7% [4], whereas a 2024 Indian series of 200 unselected bone marrow examinations found erythroid hyperplasia in 20% and acute leukaemia in only 9.5% of cases, with chronic myeloid leukaemia contributing a further 7.5% [13]. The markedly higher acute leukaemia burden observed here most likely reflects referral or case-mix bias toward a haemato-oncology population rather than a true difference in underlying population prevalence. It should therefore be interpreted with this caveat rather than as evidence of an unusually high incidence of leukaemia.

A notable and clinically relevant finding was that 65 of 89 acute leukaemia records (73.0%) had no documented lineage subtype in the source pathological opinion, although most of these records had a full immunohistochemical marker panel available. This gap between marker availability and final synoptic subclassification mirrors previously described limitations in bone marrow trephine reporting, in which morphological and immunophenotypic data are generated but not always consolidated into a WHO-aligned final diagnosis [2,11]. Because assigning a lineage using the same markers subsequently tested for diagnostic associations would have introduced circularity, we deliberately did not reclassify these records for the analyses in this study. Instead, we recommend that reporting units consider structured synoptic templates aligned with the fifth edition of the WHO classification or the International Consensus Classification frameworks to reduce this gap prospectively [7-10].

Our findings are consistent with those of Arif M et al., who similarly demonstrated the broad diagnostic utility of bone marrow morphology across a wide spectrum of haematological and selected non-haematological disorders. In their study, malignant haematological disorders predominated, with acute leukaemia being the most frequent diagnosis, further supporting the continued role of bone marrow aspiration and trephine biopsy as essential, cost-effective diagnostic tools in resource-limited settings [14].

The overall male predominance (66.9%) is consistent with several regional bone marrow biopsy series, which reported male proportions of approximately 60%-64% [4,6,13]. However, we found no statistically significant association between sex and broad diagnostic category, and the effect size was small to moderate (Cramér’s V = 0.182). Thus, although records from males were more common in most categories, there was no statistically significant evidence that the relative distribution of diagnoses differed by sex.

Age varied significantly across diagnostic categories, with the primary Kruskal-Wallis analysis showing an epsilon-squared effect size of 0.196. The classical ANOVA sensitivity analysis produced η² = 0.259. Records classified as myelodysplastic/myeloproliferative overlap and lymphoproliferative/plasma cell neoplasms involved significantly older individuals than records classified as aplastic anaemia/bone marrow failure syndrome. The MDS/MPN overlap group also involved significantly older individuals than the acute leukaemia group. This pattern is biologically expected: MDS and plasma cell neoplasms are well established as diseases of older age, as reflected in prognostic frameworks such as the Revised International Prognostic Scoring System for MDS, which was itself derived from a predominantly older adult cohort [15], whereas acquired aplastic anaemia has a bimodal but younger-skewed age distribution in several series and guideline populations [16,17]. The consistency between our findings and this established epidemiology provides some indirect support for the internal validity of our diagnostic categorisation, despite its reliance on free-text opinion mapping rather than structured coding at the source. The diagnostic groupings used for analysis were intended to facilitate statistical comparisons and do not replace formal WHO/ICC disease classification.

This study has several limitations. Because repeat examinations could not be reliably distinguished from initial diagnostic procedures within the registry, some patients may have contributed more than one record. This may have violated the assumption of independence between observations. Ethnicity was not routinely documented in the registry and therefore could not be evaluated in the present study. This was a single-centre study using retrospectively extracted registry data, with an inherent risk of misclassification arising from free-text diagnostic fields. Although this risk was mitigated through prespecified, transparent text-matching rules, residual misclassification cannot be excluded. A substantial minority of records (12.2%) had an inconclusive or deferred final opinion. Some laboratory fields also had appreciable missingness, particularly peripheral blast percentage, which was missing in 30 of 181 records (16.6%). Missing data were handled using complete-case analysis for analyses involving these fields, which may have introduced bias if the data were not missing at random. Several rare diagnostic categories contained very few records, including T-cell neoplasm (n = 1) and unspecified chronic leukaemia (n = 1), precluding meaningful subgroup inferences for these entities. Accordingly, the “other” category in the inferential tables should be interpreted cautiously. No cytogenetic or molecular data were available, limiting the ability to perform fully WHO/ICC-concordant subclassification of myeloid neoplasms, MDS, or chronic leukaemias. In particular, we could not distinguish chronic myeloid leukaemia from other chronic leukaemias in the single such record identified. Finally, as this was a descriptive cross-sectional study, no causal inferences can be drawn from the observed associations between age, sex, and diagnostic category.

Notwithstanding these limitations, the study’s strengths include a reasonably large consecutive registry series that retained nondiagnostic opinions; a transparent and reproducible data-cleaning and categorisation pipeline; and the use of exact and assumption-robust statistical methods appropriate for the sparse-cell and non-normal data encountered. These methods included a Monte Carlo approximation to the Fisher-Freeman-Halton-type exact test and the Kruskal-Wallis test with Dunn’s post hoc comparisons, rather than relying solely on the asymptotic chi-square test and classical ANOVA. The explicit avoidance of circular marker-based diagnostic assignment may also strengthen the credibility of the reported associations.

## Conclusions

In this single-centre series of 181 bone marrow examination records, acute leukaemia was the predominant diagnostic category, followed by aplastic anaemia/bone marrow failure syndrome and non-diagnostic (inconclusive/deferred) examinations. This distribution likely reflects the haemato-oncology-weighted referral base of the reporting centre rather than disease prevalence in the general population. Age varied significantly across diagnostic categories, whereas sex was not significantly associated with diagnostic category. Older age characterised the myelodysplastic/myeloproliferative overlap and lymphoproliferative/plasma cell neoplasm groups, whereas younger age characterised the aplastic anaemia/bone marrow failure syndrome group. This pattern is consistent with established disease epidemiology and provides some indirect support for the validity of the diagnostic categorisation used. A substantial proportion of acute leukaemia records lacked a fully documented lineage subtype despite the availability of immunohistochemical data, underscoring the practical need for structured, WHO/ICC-aligned synoptic reporting templates. Future prospective multicentre studies incorporating cytogenetic and molecular annotations are needed to build on these descriptive findings.
